# Stem Cell Models to Investigate the Role of DNA Methylation Machinery in Development of Neuropsychiatric Disorders

**DOI:** 10.1155/2016/4379425

**Published:** 2015-12-20

**Authors:** K. Naga Mohan

**Affiliations:** Department of Biological Sciences, Birla Institute of Technology and Science, Pilani, Hyderabad Campus, Jawaharnagar, Hyderabad 500 078, India

## Abstract

Epigenetic mechanisms underlie differentiation of pluripotent stem cells into different lineages that contain identical genomes but express different sets of cell type-specific genes. Because of high discordance rates in monozygotic twins, epigenetic mechanisms are also implicated in development of neuropsychiatric disorders such as schizophrenia and autism. In support of this notion, increased levels of DNA methyltransferases (DNMTs), DNMT polymorphisms, and dysregulation of DNA methylation network were reported among schizophrenia patients. These results point to the importance of development of DNA methylation machinery-based models for studying the mechanism of abnormal neurogenesis due to certain DNMT alleles or dysregulated DNMTs. Achieving this goal is strongly confronted by embryonic lethality associated with altered levels of epigenetic machinery such as DNMT1 and expensive approaches in developing *in vivo* models. In light of literature evidence that embryonic stem cells (ESCs) are tolerant of DNMT mutations and advancement in the technology of gene targeting, it is now possible to introduce desired mutations in DNMT loci to generate suitable ESC lines that can help understand the underlying mechanisms by which abnormal levels of DNMTs or their specific mutations/alleles result in abnormal neurogenesis. In the future, these models can facilitate development of suitable drugs for treatment of neuropsychiatric disorders.

## 1. Introduction

During postblastocyst stage of mammalian development, the embryo undergoes implantation and the cells of the inner cell mass undergo lineage specification. As a result, cells of different lineages, despite being genetically identical, express unique set of genes that are lineage-specific while keeping the nonspecific genes silent. This difference in expression patterns is achieved due to the developmental cues in the embryo but is maintained in form of distinctive epigenetic patterns of the genomes. These epigenetic marks are heritable such that they are propagated in all the daughter cells within the lineage.

DNA methylation, histone modifications, and regulatory noncoding RNAs constitute the main epigenetic marks of mammalian genomes. Of these three, DNA methylation or covalent addition of methyl group at the fifth carbon of cytosines in genomic DNA is the earliest to be reported and most well studied in terms of its establishment, maintenance, and erasure (e.g., see [1]). Most cytosines are methylated in CpG dinucleotides except in the CpG islands wherein in most cases unmethylated state has been positively correlated with gene expression [2–4]. Because of this inverse relationship with gene expression, DNA methylation and the machinery responsible for establishment/maintenance play an important role in differentiation. Whereas* de novo* methyltransferases DNMT3A and DNMT3B can impart new methylation marks on unmethylated DNA, DNMT1 is responsible for maintenance of DNA methylation throughout life [5]. These contrasting functions of* de novo* and maintenance DNMTs are reflected in their expression patterns: DNMT3A and DNMT3B are involved in epigenetic reprogramming and expressed mainly in the germline [6, 7], pluripotent, and adult stem cells [8] whereas DNMT1 is expressed in all developmental stages and in terminally differentiated tissues.

Because of their central role in creation and maintenance of DNA methylation patterns and the inverse correlation between DNA methylation and gene expression, DNMTs have been the subjects of intense research. In particular, the role of DNMTs and the influence of their dysregulation on the process of differentiation or maintenance of differentiated state are becoming unraveled in the recent past. Recent studies also indicate that dysregulation of DNMTs may be a disease-causing mechanism among patients with schizophrenia and bipolar disorders. Epigenetic mechanisms have been proposed to be involved in pathophysiology of schizophrenia and autism because of high discordance rates between monozygotic twins [9]. Although these defective epigenetic mechanisms include machinery responsible for establishment and maintenance of histone modifications as well as DNA methylation, published literature to date suggests more evidence for dysregulation of DNMTs in schizophrenia [10].

Here, I will describe the central role of DNMTs in mammalian development, the embryonic stem cell- (ESC-) based* in vitro* and animal models developed by different investigators that have helped in gaining insights into the critical role of DNMTs in pluripotency and differentiation (Table 1). I will then describe the problems associated with development of suitable animal models and propose that transgenic ESC models can help in understanding the mechanisms by which dysregulation of DNMTs or particular DNMT mutants/alleles influences neuronal differentiation. An understanding of these mechanisms would in turn help in determining the pathophysiology among those schizophrenia patients where there is dysregulation of DNMTs. Such information is also invaluable in identification of suitable drugs that help in correcting the abnormal neuronal phenotypes because of dysregulation of DNMTs.

## 2. Basic Functions of* De Novo* and Maintenance DNMTs

**Figure 1:**
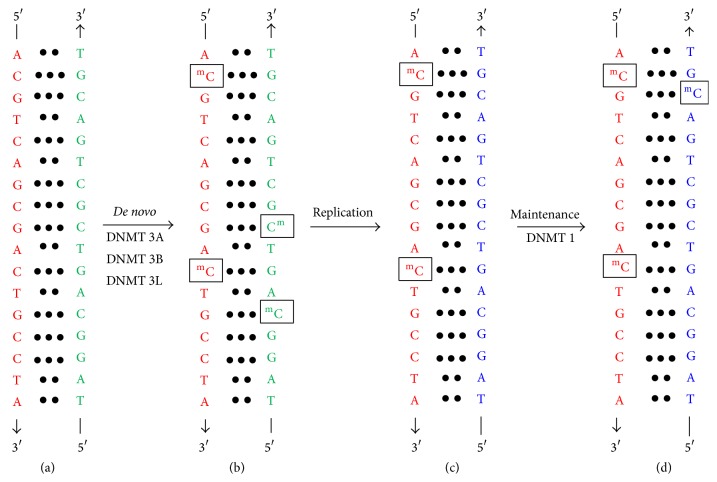
Specific roles of DNMTs in establishment and maintenance methylation in mammalian genomes. (a) Unmethylated DNA is acted upon by* de novo* methyltransferases DNMT3A and DNMT3B which methylated cytosines in the DNA molecule. The complementary strands are shown in green and red. This step also requires DNMT3L, the nonenzymatic member of DNMT family. (b) As a result of* de novo* methylation, methylated cytosines can be found in the context of both CpG and non-CpG dinucleotides. (c) After replication, the parental strand in each daughter DNA molecule serves as information for maintenance methylation. DNMT1 methylates cytosines in the daughter strand at positions that are exactly opposite to the methylated cytosines in the parent strand. (d) Because CpG dinucleotides are symmetric and exactly opposite in the daughter DNA, the maintenance methylation is highly efficient at CpG dinucleotides. Non-CpG dinucleotides do not contain cytosines at exactly the opposite positions in the two strands and therefore maintenance methylation in these dinucleotides is poor.

As mentioned above, DNMT3A and DNMT3B are the* de novo* methyltransferases whereas DNMT1 is a maintenance methyltransferase. In addition to these enzymes, DNMT3L is another DNMT family member which is catalytically inactive. DNMT3L interacts with DNMT3A and DNMT3B and is important for establishment of certain methylation marks in the germline. Once DNA methylation marks are established on unmethylated DNA, they are maintained by DNMT1 in a postreplicative manner (Figure 1 [11]). When DNA is freshly replicated, the parental and daughter strands contain methylated and unmethylated cytosines and are said to be hemimethylated. DNMT1 is proposed to “read” these methylation marks and establish methylation on the opposite cytosines in the newly synthesized complementary strand. For this reason, CpG dinucleotide is best suited for heritability of methylation marks because the complementary strand also contains the same dinucleotide in exactly the same position. Because of the lack of this symmetry, non-CpG methylation is not efficiently maintained (Figure 1).

## 3. Essential Role of DNMTs in Mammalian Development

Indications that DNMTs are absolutely essential for development came from mouse transgenic studies. ESCs in which both alleles of* Dnmt1* were disrupted showed reduction in DNA methylation levels and normal growth rates and morphology, but embryos derived from them showed stunted growth and developmental delay and did not survive after midgestation [12]. However, in mice carrying a conditional allele which results in loss of DNMT1 precursor cells of the central nervous system resulted in formation of hypomethylated neurons but these neurons quickly degenerated [13]. In subsequent studies in which the DNMT1 levels were altered, increased levels of DNMT1 also result in embryonic lethality around midgestation because of genomic hypermethylation aberrant regulation of imprinted genes (a class of genes that are expressed from only one of the two homologous chromosomes based on their parental origin) [14]. Similar to the results obtained with DNMT1 knockout embryos, absence of DNMT3A or DNMT3B affects normal development/survival. Whereas* Dnmt3B* ^*null/null*^ embryos fail to develop to term due to developmental defects,* Dnmt3A* ^*null/null*^ embryos survive to term but fail to survive beyond four weeks [15]. In contrast to lethality associated with deficiency of DNMT1 or DNMT3a or DNMT3b,* Dnm3L* ^*null/null*^ mice survive but their conceptuses have DNA methylation defects.* Dnmt3L* ^*null/null*^ females show stochastic imprinting patterns in their oocytes [16] resulting in a population of embryos that do not develop to term because of abnormal methylation patterns whereas* Dnmt3L* ^*null/null*^ males show loss of spermatogonia with wide-spread DNA methylation defects that reduce the chances of survival of their progeny [17]. Taken together, these results suggest that optimal levels of DNMTs are essential for normal development and survival.

## 4. DNMTs and Their Role Differentiation

The functional roles of DNMTs in the differentiation program were only established recently. Transgenic studies in which a catalytically inactive DNMT1 mutant was expressed in* Dnmt1* ^*c/c*^ mouse ESCs that do not produce DNMT1 showed that in absence of DNMT1 ESCs do not lose their self-renewal capacity and their ability to initiate differentiation but the mutant cells do not survive differentiation [21]. For instance, loss of DNMT1 activity has been shown to be the sole reason for the inability of the* Dnmt1* ^*c/c*^-derived neurons to survive [19]. In another set of studies, mouse ESCs were genetically engineered to produce high levels of DNMT1 from the endogenous* Dnmt1* promoter [18]. These mutant ESCs showed normal morphology and growth patterns but produced abnormal neurons that showed extensive dendritic arborization, branching, and increased levels of NR1 subunit of N-methyl D-aspartate (NMDA) receptor [23]. This study has also shown that the levels of DNMT1 are tightly regulated during differentiation and, as a result, DNMT1 is maintained at low levels in embryoid bodies and neurons. This study also indicated that downregulation of DNMT1 is a general phenomenon associated with differentiation. Consistent with this indication, two pluripotency factors OCT4 and Nanog have been shown to bind to the* Dnmt1* promoter and enable the expression of DNMT1 in ESCs [20]. It is therefore plausible to expect that downregulation of OCT4 and Nanog results in lowering of DNMT1 levels during differentiation. Effects of DNMT1 loss of function have also been investigated in hematopoietic stem cells (HSCs) wherein specific absence of DNMT1 in the hematopoietic system results in poor retention of HSCs in their niches, deficient self-renewal, and multilineage hematopoiesis. These abnormalities were accompanied by enhanced cell cycling and dysregulated mature lineage gene expression in myeloid progenitor cells [24]. Taken together, it becomes evident that DNMT1 plays a crucial role in regulating the differentiation potential of pluripotent stem cells as well as adult stem cells.

Experiments using transgenic mice lacking DNMT3A and/or DNMT3B suggest that in addition to DNMT1, DNMT3A and DNMT3B also influence differentiation. For instance, in absence of DNMT3A and DNMT3B, ESCs retain their proliferative capacity and self-renewal but progressively lose DNA methylation [15]. Interestingly, these mutant cells also progressively lose their ability to differentiate [22]. Additional roles of DNMT3A and DNMT3B on self-renewal and differentiation of hematopoietic stem cells (HSCs) became evident from patients with DNMT3A mutations. About 20% of the individuals with DNMT3A mutations present with acute myeloid leukemia and myelodysplastic syndrome, characterized by abnormal differentiation or deficiency of hematopoietic lineages. Experimental data on mutant mice in which there is a loss of DNMT3A in hematopoietic stem cells showed a block in differentiation and an expanded number of HSCs in bone marrow [25]. The molecular defect in the mutant HSCs is an increased expression of multipotency genes and a decreased expression of differentiation factors. Subsequent studies where either or both of DNMT3A and DNMT3B were conditionally knocked out in HSCs showed that double mutants show synergistic effects whereas DNMT3B mutants showed milder phenotypes [26]. In another set of experiments, DNMT3A expression in postnatal neural stem cells (NSCs) was observed to result in intergenic and gene body methylation of several regions, particularly in the gene bodies proximal to the promoters of neurogenic genes. Such methylation was shown to be required for expression of these genes to enable normal neuronal differentiation [27]. From the discussion above, a conclusion can be drawn that DNMT levels and their expression patterns determine the proliferative capacity and the differentiation potential of pluripotent and adult stem cells.

## 5. Involvement of DNMTs in Neuropsychiatric Disorders

Because of their central role in the properties of pluripotent and adult stem cells, and their differentiation potential, abnormal regulation of DNMTs or their mutations are expected to cause a broad spectrum of human disease conditions. However, their essential requirement during embryogenesis makes many of the mutations in DNMTs unrecoverable due to embryonic lethality [28]. Mutations that are mild or those that occur in specific tissue types (somatic mutations) or tissue-specific dysregulation of DNMTs may present with clinically recognizable phenotypes. The only exception seems to be DNMT3B loss of function which results in immune deficiency, centromere instability, and facial abnormalities (ICF) syndrome [29]. Among recent investigations on disorders in which epigenetic mechanisms have been implicated, a relationship between dysregulation of DNMTs and schizophrenia has been observed. For instance, elevated levels of DNMT1 were observed in the interneurons of frontal cortex of schizophrenia and bipolar patients with psychosis. This overexpression of DNMT1 was correlated with hypermethylation and downregulation of GAD67 and REELIN [30]. Both GAD67 deficiency and REELIN deficiency have been shown to cause schizophrenia-like phenotypes [31]. Since DNMT1 cannot by itself establish new DNA methylation marks, it is reasonable to also expect dysregulation of* de novo* DNMTs in the brain samples with schizophrenia. Consistent with this expectation, overexpression of DNMT3A was also observed in GABAergic neurons of schizophrenia patients [32]. Mechanistically, the relationship between overexpression of DNMT1 and schizophrenia is not fully known although a recent study showed that DNMT1 binds to the promoters of* BDNF* and GABAergic genes. This finding is in support of the hypothesis that overexpression of DNMT1 downregulates both specific GABAergic and glutamatergic genes [33]. It is important to note here that the mechanistic basis by which DNMT1 is specifically targeted to promoters such as those of* REELIN*,* BDNF*,* GAD67*, and the repressed GABAergic and glutamatergic genes is unknown. In addition to the reports that related overexpression of DNMT1 in brain samples from schizophrenia patients, certain alleles of* Dnmt1*,* Dnmt3A*, and* Dnmt3L* have been found to be associated with schizophrenia [34]. In summary, the findings described above indicate that dysregulation of DNMT expression and specific alleles of DNMTs may contribute to development of schizophrenia.

## 6. Stem Cell Models to Investigate the Role of DNMTs in Abnormal Neuronal Development

At present, there are no suitable* in vivo* models available to investigate the mechanistic basis by which overexpression of DNMTs or specific alleles of DNMTs cause phenotypes associated with schizophrenia. Animal models to investigate the mechanistic role of DNMT defects are very expensive as they require gene targeting experiments using conditional alleles that affect DNMTs in neuronal lineages. In addition, materials such as neuronal progenitors and neurons in early stages of differentiation are limited in their availability per animal and require multiple animals to be used in each study.* In vitro* models help address these problems because it is possible to genetically modify ESCs or neuronal progenitor cells, scale them up to required quantities, and induce differentiation into specific types of neurons, which in turn can be obtained in sufficient amounts. To this date,* Dnmt* ^*tet/tet*^ ESCs constitute the only cell line in which DNMT1 overexpression in ESCs results in abnormal neuronal differentiation. This transgenic cell line was obtained by inserting* tet-off* cassettes into the endogenous* Dnmt1* promoters of wild-type ESCs. As a result of transactivation mediated by the* tet-off* cassettes, the levels of DNMT1 in* Dnmt* ^*tet/tet*^ ESCs are five times higher than the wild-type cells [18]. These ESCs showed normal growth kinetics and morphological features but produced neurons that show abnormal dendritic arborization, branching as in case of patients with spontaneous limbic epilepsy, and hyperactive N-methyl-D-aspartate receptor due to overexpression of NR1 subunit [23]. Interestingly, the abnormal neurons do not overexpress DNMT1 anymore and in fact DNMT1 overexpression was lost immediately after induction of differentiation. Therefore,* Dnmt* ^*tet/tet*^ neurons appear to “remember” that DNMT1 was overexpressed in ESC stage and this memory results in abnormal neurogenesis. While there is an ESC model to investigate the role of DNMT1 overexpression in abnormal neurogenesis, the* Dnmt1* ^*tet/tet*^ neurons do not have elevated levels of DNMT1 in neurons and therefore do not represent the neurons of schizophrenia patients with psychosis.

**Figure 2:**
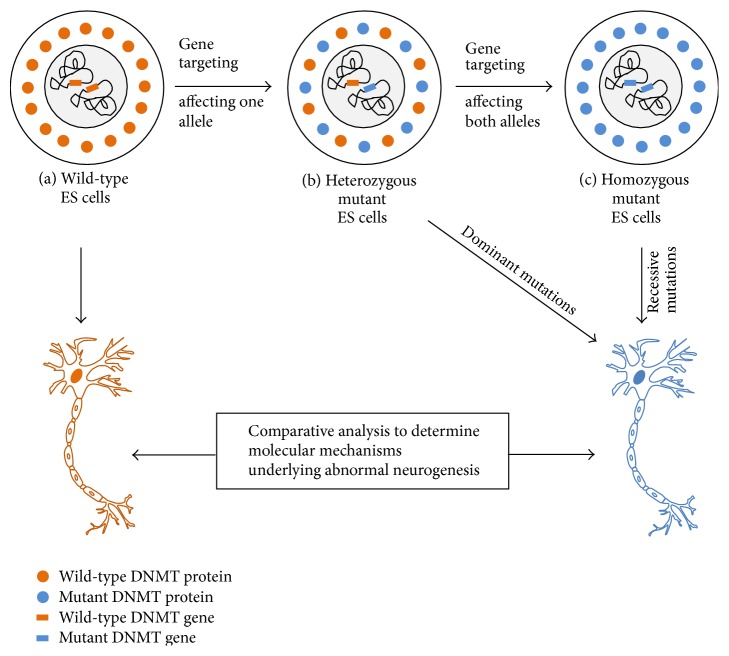
Schematic representation generation of embryonic stem cell-based models for studying the role of DNMT dysregulation on development of abnormal neuronal phenotypes. Desired mutations can be selected on the basis of literature evidence of mutations in DNMTs that are associated with neuropsychiatric disorders. Both alleles of DNMTs (*de novo *and maintenance) can be targeted to introduce desired mutations in the wild-type ES cells. First round of targeting results in heterozygotes which can then be used for a second round of targeting that results in homozygotes. Following gene targeting, homozygous ESCs (for recessive mutations) or heterozygous ESCs (for dominant mutations) can be differentiated into neurons. The resultant neurons can be used for detailed physiological and molecular-genetic studies to identify the molecular basis for abnormal neuronal phenotypes.

From the description above, it is clear that there is an absolute need for development of suitable cell-based models to study the mechanisms by which elevated levels of DNMT1 and DNMT3A, or other DNMT family members, specific disease-associated DNMT alleles cause development of abnormal neurons. These models would then give us an opportunity to test whether the resultant neurons share any molecular/phenotypic features of neurons from schizophrenia patients or from patients with other psychiatric disorders. The task of cell-based models to generate neurons that overexpress DNMTs or express specific schizophrenia-associated DNMT variants can be accomplished with relative ease because ESCs are tolerant to DNMT levels and even loss of methylation [35]. ESCs are also amenable to targeted gene modification, a method that can allow investigators to introduce specific DNMT alleles in place of wild-type alleles without altering the rest of the genome [36]. Technologies for achieving these goals are in practice for several years and are being constantly improved, especially in the recent five years. For example, CRISPR/Cas9 system is a recent technology that enables a high frequency replacement of wild-type alleles with the mutant alleles [37]. Using this system, it is possible to generate homozygous mutants that express only the mutant proteins (Figure 2). It is also possible to investigate specifically the functional role of a specific DNMT mutant by using DNMT-null ESCs to generate transgenic cell lines that only express the mutant proteins (Figure 2). To achieve constitutive overexpression of DNMTs, new generation expression vectors for ESCs are available with appropriate promoters that encode transcripts such that the transcript produced contains messages for both the gene of interest and a selection marker that confers resistance to an antibiotic such as geneticin, puromycin, and hygromycin (e.g., see [38]).

The possibility of development of ESC models offers certain advantages and disadvantages in their utility to understand the mechanistic basis by which dysregulation of DNMTs is associated with disorders such as schizophrenia.* In vitro* models offer a simple platform to investigate the disease-causing mechanisms because genetically altered ESC lines have well-defined mutations and, barring these specific changes, the remainder of the genome in each of these mutant cell lines is identical to the wild-type cells. As a result, the genotype-phenotype correlation would be clear and unequivocal. Following determination of the exact molecular defect, the* in vitro* models also become attractive tools for large-scale screening using potential drug targets to correct the abnormal neuronal phenotypes. Such high-throughput screens are difficult to perform with animal models. It is important to note that the details of affected cell types in the brain of schizophrenia patients are not well-established. In this context,* in vitro* differentiation of genetically altered ESCs might result in neuronal cell types that may not be representative of the cell types affected in schizophrenia. Although there is no complete picture, literature evidence suggests dysfunction of both GABAergic and Glutamatergic neurons in schizophrenia [39, 40]. In the light of this evidence, it is possible to “direct” differentiation of genetically modified ESCs to specifically glutamatergic and GABAergic lineages [41, 42]. In summary, genetically modified ESCs can help address the consequences of dysregulation of DNMT1 on development and function of either GABAergic or glutamatergic neurons but may not present the overall picture of abnormalities associated with a mixture of neurons and other supporting cells in the brains of schizophrenia patients.

## 7. Conclusions 

Research in the recent past by different groups has uncovered the important role of DNMTs in schizophrenia. It is also possible that, in the future, more DNMT alleles will be associated with other neuropsychiatric disorders. However, investigation of the mechanisms by which the dysregulation of DNMTs results in these disorders using animal models can be highly expensive and laborious. The availability of suitable knockout cell lines, advancements in the technology for targeted gene modifications, and constitutive expression of cDNAs in stem cells together provide a viable alternative option to animal models. Using this approach, elucidation of the mechanistic basis by which altered DNMT levels or specific DNMT alleles cause abnormal neuronal differentiation can pave the way in the future towards possible therapeutic interventions to neuropsychiatric disorders.

## Figures and Tables

**Table 1:** Effects of DNMT deficiency and overexpression in development/differentiation.

S. number	Type of the defect	Model system	Phenotype(s)	Reference
1	DNMT1 deficiency	Mouse (knockout)	Lethality at midgestation with imprinting and DNA methylation defects	[10]
		Mouse (conditional knockout in precursor cells in central nervous system)	Degeneration of neurons	[11]
		Mouse embryonic stem cells (ESCs)	Differentiated neurons do not survive, self-renewal is unaffected	[16]
		Hematopoietic stem cells (HSCs)	Poor retention in niches, deficient self-renewal, and defective hematopoiesis	[18]

2	DNMT3a deficiency	Mouse (knockout)	Failure to develop to term	[13]
		Mouse ESCs (knockout)	No effect on self-renewal, progressive loss of DNA methylation, and ability to differentiate	[19]
		Conditional knockout in hematopoietic lineage	Block in differentiation and expanded number of HSCs in bone marrow	[20]

3	DNMT3b deficiency	Mouse (knockout)	Death within four weeks after birth	[13]
		Mouse ESCs (knockout)	Self-renewal unaffected, progressive loss of DNA methylation, and ability to differentiate	[19]
		Conditional knockout in hematopoietic lineage	Defects are milder than in case of DNMT3a deficiency in HSCs; double mutants (deficient in both DNMT3A and DNMT3B) have more severe defects	[20]

4	DNMT3L deficiency	Mouse (knockout)	Females: stochastic imprinting patterns	[14]
			Males: low spermatogonia and wide-spread methylation defects	[15]

5	DNMT1 overexpression	Mouse (transgenic)	Lethality at midgestation due to imprinting defects	[12]
		Mouse ESCs (targeted knocking to increase the levels of DNMT1	Abnormal neuronal differentiation with upregulated NMDA receptor activity	[21]

6	DNMT3a and DNMT1 overexpression	Schizophrenia and bipolar patients with psychosis	Aberrant hypermethylation and downregulation of REELIN and GAD67	[22]
